# Phenotypic and Molecular Characterization of Extended-Spectrum Beta-Lactamase-Producing *Escherichia coli* in Bangladesh

**DOI:** 10.1371/journal.pone.0108735

**Published:** 2014-10-10

**Authors:** Taslima T. Lina, Bijay K. Khajanchi, Ishrat J. Azmi, Mohammad Aminul Islam, Belal Mahmood, Mahmuda Akter, Atanu Banik, Rumana Alim, Armando Navarro, Gabriel Perez, Alejandro Cravioto, Kaisar A. Talukder

**Affiliations:** 1 Centre for Food and Water Borne Diseases, International Centre for Diarrhoeal Disease Research, Bangladesh, Dhaka, Bangladesh; 2 Department of Public Health, Faculty of Medicine, Universidad Nacional Autonoma de Mexico, Mexico City, Mexico; University of Münster, Germany

## Abstract

**Background:**

Resistance to cephalosporins in Enterobacteriaceae is mainly due to the production of extended-spectrum beta-lactamase (ESBL). Little is known about ESBL-producing bacteria in Bangladesh. Therefore, the study presents results of phenotypic and molecular characterization of ESBL-producing *Escherichia coli* from hospitals in Bangladesh.

**Methods:**

A total of 339 *E. coli* isolated from patients with urinary tract and wound infections attending three different medical hospitals in urban and rural areas of Bangladesh between 2003–2007 were screened for ESBL-production by the double disk diffusion test. Isolates with ESBL-phenotype were further characterized by antibiotic susceptibility testing, PCR and sequencing of different β-lactamase and virulence genes, serotyping, and *XbaI*-macrorestriction followed by pulsed-field gel electrophoresis (PFGE).

**Results:**

We identified 40 *E. coli* with ESBL phenotype. These isolates were resistant to ceftriaxone, ceftazidime, cefotaxime, aztreonam, cefepime, and nalidixic acid but remained susceptible to imipenem. All but one isolate were additionally resistant to ciprofloxacin, and 3 isolates were resistant to cefoxitin. ESBL genes of blaCTX-M-1-group were detected in all isolates; blaTEM-type and blaOXA-1-type genes were detected in 33 (82.5%) and 19 (47.5%) isolates, respectively. Virulence genes that are present in diarrhoeagenic *E. coli* were not found. Class-1 integron was present in 20 (50%) isolates. All the ESBL-producing *E*. *coli* isolates harbored plasmids ranging between 1.1 and 120 MDa. PFGE-typing revealed 26 different pulsotypes, but identical pulsotype showed 6 isolates of serotype O25:H4.

**Conclusion:**

The prevalence of multidrug-resistant ESBL-producing *E*. *coli* isolates appears to be high and the majority of the isolates were positive for *bla*_CTX-M_. Although there was genetic heterogeneity among isolates, presence of a cluster of isolates belonging to serotype O25:H4 indicates dissemination of the pandemic uropathogenic *E. coli* clone in Bangladesh.

## Introduction

In Gram-negative bacteria, production of beta-lactamases is one of the most common mechanisms resulting in resistance to beta-lactam antibiotics. Recently, extended-spectrum beta-lactamases (ESBLs) have contributed to the dramatic increase in resistance to new generation beta-lactam agents throughout the world [1]–[4]. These enzymes are usually plasmid-encoded and have the capacity to hydrolyze many antibiotics including penicillins, cephalosporins, and aztreonam and are inhibited by clavulanic acid (CA) [2], [3]. These phenotypic characteristics facilitate the identification of ESBLs-producing organisms using routine laboratory tests such as double disk diffusion test or E-test. However, screening of ESBL-producing bacteria by antibiotic susceptibility testing is challenging [5]. Thus detection of specific genes by PCR and sequencing are commonly used for final confirmation of ESBL producers. The association of ESBLs and the presence of TEM, SHV, OXA and CTX-M-type enzymes have been investigated in many studies [6], [7]. Previous studies have shown that CTX-M-type enzymes were the most prevalent ESBLs [7], [8]. According to Lahey database in Burlington (http://www.lahey.org/Studies/) that controls numbering of new beta-lactamases, more than 130 CTX-M-type enzymes are registered, which are classified into at least five different subgroups [9]. Since ESBLs are frequently encoded by genes located on different transferable genetic elements, a variety of epidemiological situations have been documented, ranging from sporadic cases to large outbreaks [10]. Moreover, ESBL-producing strains are often resistant to antibiotics of other classes (sulfonamides, aminoglycosides, quinolones) which complicates the treatment strategies in many hospitalized patients [11].

Although ESBLs have been detected in a many different of Gram-negative bacteria, *Klebsiella pneumoniae* and *Escherichia coli* remain the major ESBL-producing organisms worldwide [6], [12]. In addition, ESBL-producing organisms are frequently the cause of urinary tract infections (UTI) and also in surgical wound infections [13], [14]. There is one report from Bangladesh where they found 43.2% of *E. coli* and 39.5% of *K. pneumoniae* isolated from a hospital were ESBL producers [15]. However, there is a lack of information on molecular characterization of ESBL-producing organisms isolated in Bangladesh. Therefore, the objectives of the present study were the examination of presence of beta-lactamase genes in *E. coli* from different hospitals in Bangladesh.

## Materials and Methods

### Bacterial isolates

Between June 2003 and December 2007 we collected 339 of *E. coli* isolates from in-patient and out-patient departments of three hospitals in Bangladesh: The Bangabandhu Sheikh Mujib Medical University (BSMMU) in Dhaka; The Sylhet Medical College Hospital (SMCH) in Sylhet and The International Centre for Diarrhoeal Disease Research, Bangladesh (icddr,b) in Dhaka. These *E. coli* were isolated from either urine samples or surgical wound swabs from 339 patients with symptoms of UTI (n = 274) and wound infection (n = 64). All urine samples were obtained from outpatient departments and all surgical wound swab samples were from inpatient department of respected hospitals. *E. coli* were isolated and identified by using standard microbiological and biochemical methods [16]. These isolates were grown in trypticase soy broth containing 0.3% yeast extract (TSBY) and stored at −70°C after addition of 15% glycerol.

Since all isolates were collected anonymously no ethical approval was necessary for this study.

### Antimicrobial susceptibility testing

Bacterial susceptibility to antimicrobial agents was determined by the disk diffusion method following the guidelines of Clinical and Laboratory Standards Institute (CLSI) using commercially available antibiotic disc (Oxoid, Basingstoke, United Kingdom). The antibiotic discs used in this study were ceftriaxone (30 µg), ceftazidime (30 µg), cefotaxime (30 µg), cefepime (30 µg), cefoxitin (30 µg), aztreonam (30 µg), imipenem (10 µg), ciprofloxacin (5 µg) and nalidixic acid (30 µg). *E. coli* (ATCC 25922) and *Staphylococcus aureus* (ATCC 25923) were used as control strains for susceptibility test [17].

All 339 *E*. *coli* isolates were screened for the production of ESBLs by using the double disk diffusion test as described previously [18], with some modifications [4]. After overnight incubation at 37°C, any enhancement of the zone of inhibition between a beta-lactam disk and that containing the beta-lactamase inhibitor was indicative of the presence of an ESBL.

### Detection of beta-lactamase and virulence genes

PCR screening for presence of different beta-lactamase genes (*bla*TEM-type, *bla*SHV-type, *bla*OXA-1-type, *bla*CTX-M-1-group, *bla*CTX-M-2-group, *bla*CTX-M-8-group, *bla*CTX-M-9-group, *bla*CTX-M-15) and class 1 and class 2 integrons were performed as described previously [12], [19]–[22]. The primer sequences used for the detection of virulence genes, such as, invasive plasmid antigen (*ipa*H), heat labile toxin (*lt*), heat stable toxin (*st*), attaching and effacing phenotype (*eae*), invasion associated locus (*ial*), shiga toxin 1 (*stx*_1_), shiga toxin 2 (*stx*_2_) and aggregative property (*e_Agg_*) have been described in previous studies [19], [23], [24].

### Amplification of qnrA, qnrB, and qnrS

Multiplex PCR of 40 ESBL isolates were performed to detect the *qnr*A, *qnr*B, and *qnr*S according to the procedure described earlier [25]. The primer sequences are 5′-AGAGGATTTCTCACGCCAGGA-3′ and 5′-GGCTGGCCGATTATGATTGGT- 3′for *qnr*A, 5′-GGCTGGCCGATTATGATTGGT-3′ and 5′-CGCGTGCGATGAGATAACC-3′ for *qnr*B, 5′-TGCCACTTTGATGTCGCAGAT-3′ and 5′-CGCACGGAACTCTATACCGTAG-3′ for *qnr*S.

### DNA sequence analysis

Chromosomal DNA from representative strains was prepared and purified by procedures described previously. Sequencing was performed to identify specific TEM and OXA type ESBL genes. Sequencing of *gyr*A and *par*C genes were performed in the *qnr* positive ESBL isolates according the procedure described elsewhere [25]–[28]. After PCR, the amplicons were purified with the GFX PCR DNA and gel band purification kit (Amersham Pharmacia, USA), and sequenced using the dideoxy-nucleotide chain termination method with an ABI PRISM BigDye Terminator Cycle Sequencing Reaction kit (Perkin-Elmer Applied Biosystems, Foster City, California) on an automated sequencer (ABI PRISM 310). The chromatogram sequencing files were inspected using Chromas 2.23 (Technelysium, Queensland, Australia), and contigs were prepared using SeqMan II (DNASTAR, Madison, WI). Nucleotide and protein sequence similarity searches were performed using the National Center for Biotechnology Information (NCBI, National Institutes of Health, Bethesda, MD) BLAST (Basic Local Alignment Search Tool) server on GenBank database, release 138.0 [29]. Multiple sequence alignments were developed using CLUSTALX 1.81 [30]. Sequences were manually edited in the GeneDoc version 2.6.002 alignment editor. Sequences of OXA and TEM genes reported in this paper were submitted to the Genbank using the National Center for Biotechnology Information (NCBI, Bethesda, MD) Sequin, version 7.77 under the accession number EU752482-86 and EU752487-91, respectively.

### Bacterial strain typing

#### Serotyping

All *E. coli* with ESBL phenotype were serotyped by agglutination assays using 96-well microtitre plates with rabbit serum (SERUNAM) obtained against 187 somatic antigens and 53 flagellar antigens for *E. coli*, and against 45 somatic antigens for *Shigella* species described earlier [31].

#### Isolation of plasmid DNA

Plasmid DNA was prepared according to the alkaline lysis method of Kado and Liu [32] with some modifications as described previously [33]. The molecular mass of the unknown plasmid DNA was assessed by comparing with the mobility of the known molecular weight plasmids. Plasmids present in strains *E. coli* PDK-9, R1, RP_4_ and V517 described previously were used as molecular mass standards [33].

#### Pulsed-field gel electrophoresis (PFGE)

Intact agarose-embedded chromosomal DNA from clinical isolates of *E. coli* was prepared and PFGE was performed using the contour-clamped homogeneous electric field (CHEF-Mapper) apparatus from Bio-Rad Laboratories (Richmond, CA, USA) according to the procedures described elsewhere [33]. Genomic DNA was digested with *Xba*I restriction enzyme (Gibco-BRL). The restriction fragments were separated by using CHEF-mapper system apparatus in 1% pulsed-field certified agarose in 0.5× TBE buffer. The DNA size standards used was the *Salmonella enterica* serovar Braenderup (H9812) ranging from 20.5 to 1,135 kb [34]. Banding patterns were analyzed according to the established criteria reported elsewhere [35].

## Results

Of the 339 *E. coli* isolates, 11.8% (n = 40) were suspected to be ESBL producers using double disk diffusion test, of which 8.4% (23/274) were isolated from urine and 26.5% (17/64) from surgical wound samples. All *E. coli* with ESBL phenotype (n = 40) isolates were resistant to 3^rd^ generation cephalosporins. These isolates were also resistant to monobactams, such as, aztreonam and cefepime (4^th^ generation cephalosporin). Only 3 (7.5%) isolates were resistant to cefoxitin (2^nd^ generation cephalosporins). In addition, all isolates except for one were resistant to ciprofloxacin and all were resistant to nalidixic acid, whereas none of the isolates were resistant to imipenem.

PCR for beta lactamase specific genes showed that all the 40 isolates contained the *bla*_CTX-M-1_ group and *bla*_CTX-M-15_ genes. In addition, *bla*_OXA-1_ type and *bla*_TEM_-type, genes were present in 33 (82.5%) and 19 (47.5%) isolates, respectively (Table 1). Class 1 integron gene was detected in 20 (50%) isolates. Furthermore, we also examined for the presence of different virulence genes that are usually present in diarrhoeagenic *E*. *coli* isolates. None of these isolates were positive for any virulence genes such as, *lt*, *st*, *eae*, *ial*, *stx*_1_, *stx*_2_ and *e_Agg_*.

**Table 1:** Characterization of ESBL-producing *E. coli*.

Strain ID	Year of Isolation	Clinical Diagnosis	Hospital	In/Out patient	Sample	TEM type^a^	OXA type^a^	CTX-M-group 1	CTX-M-15	*qnr*S	*int*-1	Plasmid pattern	Serotype	PFGE pattern
KE1	2003	UTI	BSMMU	Out	U	+	+	+	+	-	+	P1	Atypical	N
KE2	2004	WI	BSMMU	In	SWS	+	+	+	+	-	+	P13	O64:H28	J
KE3	2004	WI	BSMMU	In	SWS	+	+	+	+	-	+	P5	Atypical	B
KE4	2003	UTI	BSMMU	Out	U	+	+	+	+	-	+	P3	O1:H30	G
KE5	2004	UTI	BSMMU	Out	U	+	+	+	+	-	+	P10	O1:H6	I
KE6	2004	UTI	BSMMU	Out	U	+	+	+	+	-	+	P3	O8:H9	M
KE7	2004	UTI	BSMMU	Out	U	+	+	+	+	-	+	P3	O101:H^-^	F
KE8	2005	UTI	SMCH	Out	U	+	+	+	+	-	-	P21	O20:H^-^	C
KE9	2005	WI	BSMMU	In	SWS	+	+	+	+	-	-	P11	Atypical	E
KE10	2005	WI	BSMMU	In	SWS	+	+	+	+	-	-	P14	Atypical	T
KE11	2005	WI	BSMMU	In	SWS	+	+	+	+	-	-	P12	O8:H49	Y
KE12	2005	WI	BSMMU	In	SWS	+	+	+	+	-	-	P7	O102:H4	P
KE13	2005	WI	BSMMU	In	SWS	-	+	+	+	-	-	P15	O102:H6	U
KE14	2006	WI	BSMMU	In	SWS	-	+	+	+	-	+	P1	Atypical	L
KE15	2006	WI	BSMMU	In	SWS	-	+	+	+	-	+	P1	O25:H4	A
KE16	2006	WI	BSMMU	In	SWS	-	+	+	+	+	+	P24	O102:H6	B
KE17	2006	WI	BSMMU	In	SWS	-	+	+	+	+	+	P5	O25:H4	A
KE18	2005	UTI	BSMMU	Out	U	-	+	+	+	-	+	P17	O25:H4	Z
KE19	2005	UTI	BSMMU	Out	U	-	+	+	+	-	+	P19	O153:H6	A
KE20	2005	UTI	SMCH	Out	U	-	+	+	+	+	-	P8	O8:H9	H
KE21	2005	UTI	SMCH	Out	U	-	+	+	+	-	-	P2	O25:H9	A
KE22	2006	UTI	SMCH	Out	U	-	+	+	+	-	-	P2	O25:H4	A
KE23	2006	UTI	SMCH	Out	U	-	+	+	+	-	-	P2	O25:H4	A
KE24	2006	UTI	SMCH	Out	U	-	+	+	+	-	-	P2	O25:H4	A
KE25	2006	WI	BSMMU	In	SWS	-	+	+	+	-	-	P8	O25:H4	S
KE26	2006	WI	BSMMU	In	SWS	-	+	+	+	-	-	P1	Atypical	V
KE27	2006	WI	BSMMU	In	SWS	-	+	+	+	-	-	P6	O132:H25	C
KE28	2007	WI	BSMMU	In	SWS	-	+	+	+	-	-	P4	Atypical	A
KE29	2006	UTI	SMCH	Out	U	+	-	+	+	-	-	P7	O20:H^-^	C
KE30	2006	UTI	BSMMU	Out	U	+	-	+	+	-	-	P6	Atypical	Q
KE31	2007	UTI	BSMMU	Out	U	+	-	+	+	-	-	P18	Atypical	B
KE32	2007	WI	BSMMU	In	SWS	-	-	+	+	-	-	P25	O102:H6	X
KE33	2007	WI	BSMMU	In	SWS	+	-	+	+	-	+	P4	O146:H31	B
KE34	2007	UTI	icddr,b	Out	U	+	-	+	+	+	+	P22	Atypical	R
KE35	2007	UTI	icddr,b	Out	U	+	+	+	+	-	+	P16	Atypical	W
KE36	2007	UTI	icddr,b	Out	U	-	+	+	+	+	+	P23	Atypical	O
KE37	2007	UTI	icddr,b	Out	U	+	+	+	+	-	+	P20	O25:H4	A
KE38	2007	UTI	icddr,b	Out	U	-	+	+	+	-	+	P9	O1:H6	D
KE39	2007	UTI	icddr,b	Out	U	-	+	+	+	-	+	P9	O153:H6	D
KE40	2007	UTI	icddr,b	Out	U	-	-	+	+	-	-	P10	Atypical	K

Abbreviations: UTI: Urinary Tract Infection, WI: Wound Infection. U: Urine, SWS: Surgical Wound Swab, ^a^ Sequencing performed on representative strains, (+) indicates presence and (-) indicates absence.

Of 40 ESBL isolates, 5 were found to carry the *qnr*S gene. In order to understand the fluoroquinolone resistance mechanism of 5 *qnr*S positive ESBL isolates, we sequenced the *gyr*A (gyrase A, a subunit of topoisomerase II) and *par*C (subunit of topoisomerase IV) genes and identified double mutations in *gyr*A (Ser^83^ → Leu, Asp^87^ → Asn) and a single mutation in *par*C (Ser^80^ → Ile) genes. Sequencing of *bla*_OXA_-_1_ type and *bla*_TEM_-type genes from 5 representative isolates identified these genes as beta-lactamase genes *bla*_OXA-1_ and *bla*_TEM-1_ respectively.

All 40 isolates yielded heterogeneous plasmid pattern ranging in size from 1.1 to 120 MDa. Middle ranged plasmid (plasmids with a size between 30 MDa and 90 MDa) was found to be present in 65% (n = 26) isolates, while 45% (n = 18) isolates harbored 120 MDa plasmid. Plasmids ranged in size from 53 to 120 MDa were detected in all the ESBL-producing *E. coli* (Table S1).

**Figure 1:**
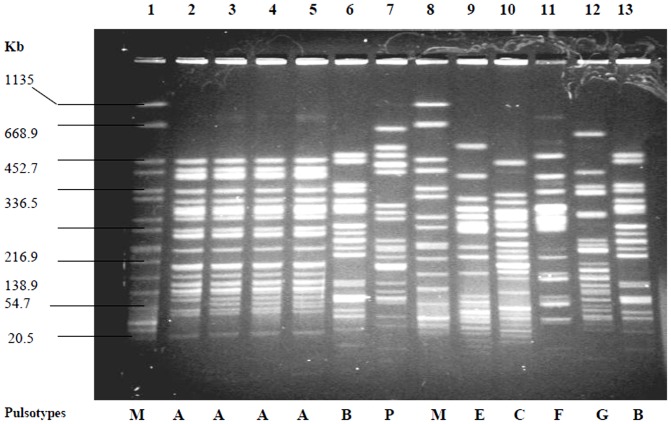
PFGE banding patterns of *Xba*I-digested chromosomal DNA of representative ESBL-producing *E. coli* isolates. Lane 1, *Salmonella enterica* serovar Braenderup (H9812) (marker); Lanes 2-5, PFGE type A; Lane 6, PFGE type B; Lane 7, PFGE type P; Lane 8, *Salmonella enterica* serovar Braenderup (H9812) (marker); Lane 9, PFGE type E; Lane 10, PFGE type C; Lane 11, PFGE type F; Lane 12, PFGE type G; Lane 13, PFGE type B. Four isolates belonged to PFGE pattern A were isolated from UTI patients attending SMCH hospital.

PFGE analysis revealed 26 pulsotypes, of which 9 isolates were grouped in type A, 4 in type B, 3 in type C and 2 in type D (Figure 1) and the remaining 22 isolates yielded an individual pulsotype (Table 1). Of these 40 isolates, the predominant serotype was O25:H4 (n = 8, 20%), followed by O102:H6 (n = 3, 7.5%), O1:H6 (n = 2, 5%), O8:H9 (n = 2, 5%), O20: H- (n = 2, 5%), and O153:H6 (n = 2, 5%). Of the remaining 21 isolates, 8 (20%) belonged to a single serotype and 13 (32.5%) were atypical (Table 1). The predominant PFGE type A was mostly found in serotype O25:H4 isolates (6/8) and PFGE type B was in atypical (2/13) whereas PFGE type C is present in serotypes O20:H- (2/2) and O132:H25 (1/1). PFGE type A was identified in different hospitals and locations but PFGE type B and C were only found in BSMMU in Dhaka (Table 1).

## Discussion

The emergence of ESBL-producing organisms has been reported in many parts of the world [1–4). In 2004, a study in Bangladesh showed that high prevalence (43.2%) of ESBL-producing *E. coli* was found in an urban Hospital in Dhaka [15]. The present study showed that around 12% of *E. coli* isolates obtained from patients with UTI and wound infections were ESBL-producers. The difference in prevalence between these two studies would be due to the sampling from different hospitals and from different geographic locations, which is the case for this study (Table 1) whereas in the previous study, samples were collected only from single hospital setting. However, occurrence of ESBL-producing bacteria in clinical specimens at present might be different than the rate that we found in this study as we analyzed the isolates collected during the period 2003–2007.

In present study, among 40 ESBL-producing *E*. *coli* strains, O25:H4 was identified as the predominant serotype (n = 8, 20%). Importantly, a particular clone detected by multilocus sequence typing (MLST) in this serogroup (O25:H4-ST131) of ESBL-producing *E*. *coli* has disseminated in many countries in Europe and in Asia [36], [37]. O25:H4 is mainly associated with production of CTX-M-15. Ceftazidime resistance indicated presence of CTX-M-15 in our O25:H4 isolates and we confirmed the presence of CTX-M-1group and CTX-M-15 in all isolates. Based on our results it is highly probable that the ESBL-producing clone *E. coli* O25:H4-ST131 is present in our study collection from different hospitals. Thus, further analyses especially MLST are needed to investigate the dissemination of this clone in future.

The antibiotic susceptibility data indicated that ESBL-producing *E. coli* isolates acquired resistance mechanisms against different classes of antibiotics that are frequently used for empirical treatment of UTIs and other extra intestinal infections. Interestingly, all 40 ESBL-producing isolates except for one were resistant to ciprofloxacin (n = 39, 97.5%), of which 5 isolates were positive for plasmid mediated quinolone resistance gene *qnr*S along with mutations in gyrA/parC genes demonstrating underlying causes of resistance to fluoroquinolones. In our previous study, we found ciprofloxacin resistance ESBL-producing *E. coli* were positive for *qnr*S gene [38] and similar findings were found in a study from Spain in 2008 [27]. This may reflect the indiscriminate and inappropriate use of antibiotics in Bangladesh where these drugs are often sold over the counter without a physician's prescription [15]. All ESBL-producing *E*. *coli* isolates were susceptible to carbapenems, the current drug of choice for the treatment of patients infected with multidrug-resistant ESBL-producing bacteria.

Similar to other studies within or outside of Bangladesh, we found that all clinical isolates were positive for CTX-M-1 group and CTX-M-15 specific genes [7], [39]. Moreover, previous reports from Bangladesh showed a high prevalence of CTX-M-15 group in ESBL producing *E. coli* isolated from household tap water and pigeons [37], [38], [40]. The CTX-M type beta-lactamases represent a rapidly emerging group worldwide, which have been found predominantly in *Enterobacteriaceae*, particularly in *E. coli*, *Klebsiella. pneumoniae*, *Proteus mirabilis* and *Salmonella Typhimurium* [2], [8], [41]–[43].

Sequence analysis of PCR product of TEM-1 and OXA-1 genes from selected isolates showed that all strains were identical to TEM-1 and OXA-1 which correlates with other studies [44], [45]. ESBL genes located on integrons-like structures are being increasingly reported worldwide [10], [41]. Hence, we examined the presence of class 1 and class 2 integrons. In our study, 50% (n = 20) of *E. coli* isolates harbored class 1 integron while none of the isolates contained class 2 integron as found in previous studies [46], [47]. However, further study is needed to investigate the association between class 1 integron production and dissemination of ESBL type genes in *E*. *coli* from Bangladesh.

Interestingly, we identified no virulence genes (*ipa*H, *ial*, *lt*, *st*, *eae*, *stx*_1_, *stx*_2_, and *e_Agg_* genes). These virulence genes are normally present in diarrhoeagenic *E. coli* which cause gastrointestinal infections [48]. Therefore, it can be explained that *E. coli* causing extra-intestinal infections such as UTI and surgical wound infection might have a different set of virulence factors than that of *E. coli* causing intestinal infection.

Apart from serotyping, plasmid profiles of all 40 isolates were compared between each other to demonstrate variation and epidemiological linkage among ESBL producing isolates. All isolates contained multiple plasmids ranging in size from 1.1 to 120 MDa and plasmid patterns of these isolates were heterogeneous (Table S1), which correlates with previous findings [38]. Plasmids ranged in size either 53 or>120 MDa or both were detected in all the ESBL-producing *E. coli* strains supporting previous findings that plasmids carrying beta-lactamase genes are ranged in size from 53 to 200 MDa [49]. Due to possible occurrence of intrinsic *bla*_CTX-M_ genes further analyses are needed to determine the exact location of the *bla*_CTX-M_ genes.

PFGE profile analysis showed heterogeneity among majority of isolates except for a few that could be clustered into a single PFGE type (9 isolates in type A, 4 isolates in type B, 3 isolates in type C and 2 isolates type D) (Table 1). It is interesting to note that isolates grouped into PFGE pattern A were mostly belonged to serotype O25:H4 (6/8) of which 4 were from patients with UTI at outpatient department and the remaining 2 were from patients with wound infections attending hospitals. These isolates were isolated in 2006 from three different hospitals which are geographically distant from each other. However, PFGE analysis of the majority of isolates demonstrated a low clonal relationship (26 clones/40 isolates), similar findings reported in previous studies [6], [50]. These findings can be explained by the fact that ESBL production might be a consequence of horizontal gene transfer between bacteria rather than the spread of specific bacterial clones.

In conclusion, high prevalence (11.8%) and presence of variety of beta-lactamase genes in ESBL-producing *E*. *coli* possibly reflects the overuse and misuse of antibiotics in Bangladesh and severely limits the therapeutic options in Bangladesh. Surveillance of multidrug-resistant Gram negative bacteria causing intestinal and extra-intestinal infections is essential to guide the empirical treatment strategies for these infections in Bangladesh. Nevertheless, this is the first effort to characterize the ESBL-producing clinical isolates in Bangladesh using molecular techniques.

### Ethical statement

The Enteric and Food Microbiology laboratory of icddr,b is a research laboratory where all anonymous samples were tested for respective pathogens, and for this present analysis anonymous samples were collected from different hospitals. So no ethical approval was needed.

## Supporting Information

Table S1 Plasmid profile analysis of ESBL-producing *E*. *coli.*
(DOC)
