# Supplementary material for: Phenotypic and Molecular Characterization of Extended-Spectrum Beta-Lactamase-Producing Escherichia coli in Bangladesh
Source: PLoS One. 2014 Oct 10;9(10):e108735. doi: 10.1371/journal.pone.0108735 (PMC4193765; doi:10.1371/journal.pone.0108735)
Supplement: Table S1 — Plasmid profile analysis of ESBL-producing E. coli. (DOC) [file pone.0108735.s001.doc]

Supplementary Table: Plasmid profile analysis of ESBL-producing *E*. *coli*

| Hospital | Plasmid Pattern | Plasmid size in MDa | No. of isolates |
| --- | --- | --- | --- |
| BSMMU | P1 | 120, 3.4 | 4 |
| SMCH | P2 | 120, 2.7, 2.6 | 4 |
| BSMMU | P3 | 120 | 3 |
| BSMMU | P4 | 120, 35.8, 28 | 2 |
| BSMMU | P5 | 120, 70-50 | 2 |
| BSMMU | P6 | 54, 3.7, 3.1 | 2 |
| BSMMU | P7 | 90-70 | 2 |
| BSMMU | P8 | 90, 1.4 | 2 |
| icddr,b | P9 | 76, 2.7, 2.0 | 2 |
| BSMMU, icddr,b | P10 | 74,54, 2.0 | 2 |
| BSMMU | P11 | 53, 3.4, 1.6 | 1 |
| BSMMU | P12 | 90, 7.8 | 1 |
| BSMMU | P13 | 90, 31, 2.1 | 1 |
| BSMMU | P14 | 90, 2.0 | 1 |
| BSMMU | P15 | 90, 20, 14, 3.4, 1.4 | 1 |
| icddr,b | P16 | 120, 2.0 | 1 |
| BSMMU | P17 | 120, 3.0 | 1 |
| BSMMU | P18 | 90, 54, 2.2 | 1 |
| icddr,b | P19 | 71, 2.1 | 1 |
| icddr,b | P20 | 120, 2.8, 2.2 | 1 |
| SMCH | P21 | 90, 33 | 1 |
| icddr,b | P22 | 62, 2.9, 2.7 | 1 |
| icddr,b | P23 | 62, 3.2, 2.6, 2.3, 1.1 | 1 |
| BSMMU | P24 | 53, 5.1, 2.3, 2.1 | 1 |
| BSMMU | P25 | 71, 2.2, 1.6 | 1 |
